# The clinical characteristics of acute cerebral infarction patients with thalassemia in a tropic area in China

**DOI:** 10.1515/tnsci-2022-0290

**Published:** 2023-06-20

**Authors:** Liyuan Liu, Xinyu Ben, Chang Li, Jiaqi Liu, Lin Ma, Xiaoping Liao, Qin Zou, Qifu Li

**Affiliations:** Department of Neurology, The First Affiliated Hospital, Hainan Medical University, Haikou 570102, China; The First Affiliated Hospital, Henan University, Kaifeng 475000, China; Key Laboratory of Brain Science Research and Transformation in Tropical Environment of Hainan Province, Hainan Medical University, Haikou 570102, China; Department of Medical Psychology, The First Affiliated Hospital, Hainan Medical University, Haikou 570102, China

**Keywords:** acute cerebral infarction, thalassemia, clinical features, tropic area, iron-deficiency anemia

## Abstract

This study aimed to explore the clinical characteristics of acute cerebral infarction (ACI) patients with thalassemia through the analysis of clinical data. Adult patients with ACI who were admitted to the First Affiliated Hospital of Hainan Medical College, the Second Affiliated Hospital of Hainan Medical College, Hainan Provincial People’s Hospital, and the Department of Neurology of Haikou People’s Hospital from January 2008 to December 2018 were enrolled. According to the eligibility criteria, 183 ACI patients were examined, of whom there were 33 cases with thalassemia, 50 cases with iron-deficiency anemia (IDA), and 100 non-anemic cases. Laboratory data, including platelet count, homocysteine count, and hemoglobin level, were collected. Besides, the results of auxiliary examinations, such as brain magnetic resonance imaging or computed tomography, carotid ultrasound, electrocardiogram, and cardiac color ultrasound, were collected. Baseline clinical data (e.g., history of smoking and drinking) were acquired. The clinical characteristics were compared and analyzed among the three groups. There were more female ACI patients with thalassemia than male ones. Furthermore, lesions in the thalassemia and IDA groups were mainly located in the region from the corona radiata and the centrum semiovale, in which multiple small infarcts were dominant. In the non-anemia group, patients’ lesions were mainly found in the basal ganglia area, and single small infarcts had the highest proportion.

## 1 Introduction

Acute cerebral infarction (ACI), also known as ischemic stroke, refers to the sudden decrease or interruption of blood supply to the brain cells, resulting in hypoxia of local tissue and depletion of energy. Eventually, cells in this area lose their activity and undergo tissue necrosis, further resulting in the loss of nerve function. ACI is the most common type of stroke, accounting for about 60–80% of cases with stroke [1]. ACI has a rapid onset, and high rates of morbidity, mortality, and recurrence, and has become the first cause of urban-related deaths [2]. Expensive treatment costs and a high disability rate make patients lose the labor force, which undoubtedly poses a huge burden to patients and their families, and negatively influences the economy. At present, ACI is not only a main clinical challenge in China, but also several countries are developing preventive and therapeutic measures to decrease the risk of ACI. In spite of great efforts made in disease prevention and treatment in China, the incidence rate of ACI is still noticeable. Thalassemia is an inherited autosomal recessive disease with microcytic hypochromic anemia resulting from reduced or absent synthesis of one or more of the globin chains of hemoglobin (Hb). Thalassemia is especially prevalent in Mediterranean countries, Southeast Asia, Africa, Middle East, and in the Indian subcontinent [3]. Some scholars analyzed blood samples of 8,868 local residents in Hainan province (China), and the results showed that there were 2,547 thalassemia-positive cases, accounting for 28.7%, which was slightly more prevalent in women than in men, and significantly higher than the national average of 2.64% [4]. Regarding the pathogenesis, thalassemia can be divided into α, β, αβ, and δ types, of which α- and β-thalassemia are more common. Clinically, according to the severity of anemia, thalassemia is mainly divided into mild, intermediate (thalassemia intermedia, TI), and major thalassemia (thalassemia major, TM) [5]. With the improvement in medical services, the average life expectancy of thalassemia patients has been prolonged, while relevant complications have also emerged. The underlying etiology in such patients has not been fully elucidated, while it could be related to hypercoagulability, age, iron overload, and anemia [6]. The present study aimed to analyze the clinical characteristics of ACI patients with thalassemia, in order to explore the underlying mechanism, and to provide a reliable reference for diagnosis and treatment of ACI patients complicated with thalassemia.

## 2 Methods

### 2.1 Participants

ACI patients with thalassemia and iron-deficiency anemia (IDA) who were admitted to the First Affiliated Hospital of Hainan Medical College, the Second Affiliated Hospital, the Affiliated Provincial People’s Hospital, and the Department of Neurology of Haikou People’s Hospital (Haikou, China) from January 2008 to December 2018 were included. ACI patients with and without anemia were allocated to thalassemia group (Tha group), IDA group, and non-anemia (NA) group.

1. Inclusion criteria
  The following were the inclusion criteria: Patients who were aged ≥18 years old; patients who met the diagnostic criteria for cerebral infarction proposed by the Fourth National Cerebrovascular Disease Conference of the Chinese Medical Association, and confirmed by computed tomography (CT) or magnetic resonance imaging (MRI) of head; acute onset (duration of disease onset would be less than 72 h); patients in the Tha group met the diagnostic criteria for thalassemia or thalassemia screening test; patients in the IDA group met the criteria for IDA.
2. Diagnostic criteria
  Patients in the NA group met the peripheral blood Hb level ≥ 120 g/L (male) and ≥ 110 g/L (female).
3. Exclusion criteria

The exclusion criteria were as follows: Patients with cerebral infarction with large artery atherosclerosis and small artery occlusion; patients with cardiogenic cerebral embolism or cerebral embolism caused by other causes; patients with other anemia-associated diseases; patients with malignant tumors and multiple-organ failure; patients who received intravenous thrombolysis or interventional therapy; patients with incomplete data.

### 2.2 Clinical data acquisition

Gender and age of all patients were recorded.

In addition, Hb level, platelet count, cholesterol level, and homocysteine (Hcy) level were recorded, and according to the degree of anemia, patients were assigned into mild-anemia group (male: <120 g/L, female <110 g/L), moderate-anemia group (90–60 g/L), and severe-anemia group (<60 g/L).

In the present study, paresthesia, movement disorder, speech disturbance, disturbance of consciousness, ataxia, cognitive dysfunction, mental disorder, and visual disturbance were used as observational indicators.

Eight common controllable risk factors for cerebral infarction, such as hypertension, hyperlipidemia, diabetes, high Hcy level, history of smoking, history of drinking, carotid arteriosclerosis or stenosis, intracranial arteriosclerosis and stenosis, were used.

### 2.3 Imaging data acquisition

To localize the infarct lesions, the following brain regions were considered: lobes, corpus callosum, basal ganglia, corona radiata and centrum semiovale, brainstem, and cerebellum; according to the size of the infarct lesions, they were divided into small infarct lesions (≤3 cm), medium infarct lesions (3 cm < lesion ≤ 5 cm), and large infarct lesions (>5 cm) [7]; lesions were divided into multiple infarct lesions and single infarction lesions according to the type of attack; the location, size, and number of lesions were recorded and analyzed. In addition, the vascular distribution of the lesions is divided into anterior circulation, posterior circulation, and anterior and posterior circulation according to imaging findings; the hardening and stenosis of large arteries were also recorded. All patients were scanned by MRI or CT, including T1-weighted imaging (T1WI), T2WI, magnetic resonance angiography (MRA), diffusion-weighted imaging (DWI), and fluid-attenuated inversion recovery (FLAIR) sequences.

### 2.4 Statistical analysis

Statistical analysis was performed using the SPSS 21.0 software (IBM Corp., Armonk, NY, USA). Normally distributed quantitative data were expressed as mean ± standard deviation, and abnormally distributed quantitative data were expressed as median and interquartile range. Qualitative data were presented as number and frequency. The Chi-square test was used for the analysis of categorical variables. Fisher’s exact test was utilized for the expected frequency count of <5. Student’s *t*-test was used to analyze statistically significant differences between the two groups. *P* < 0.05 was considered statistically significant.


**Ethical approval:** The research related to human use has been complied with all the relevant national regulations, institutional policies and in accordance the tenets of the Helsinki Declaration, and has been approved by the authors’ institutional review board or equivalent committee. This research was approved by the Ethics Committee of the First Affiliated Hospital of Hainan Medical university No. 2018018 (Science Research).
**Informed consent:** Informed consent has been obtained from all individuals included in this study.

## 3 Results

### 3.1 Clinical and demographic data

A total of 33 cases were involved in the Tha group, including 13 males and 20 females. There were 50 cases in the IDA group, including 28 males and 22 females. A total of 100 cases were included in the NA group (male [*n* = 67] vs female [*n* = 33]).

The gender-based difference between the Tha and NA groups was statistically significant (*P* < 0.05); the gender-based difference between the Tha and IDA groups was not statistically significant (*P* > 0.05). There was no statistically significant difference in mean age among the Tha, NA, and IDA groups (*P* > 0.05). Table 1 compares demographic data among the three groups.

**Table 1:** Comparison of demographic data among the three groups

Variables	Tha (*n* = 33)	IDA (*n* = 50)	NA (*n* = 100)	*F*/*χ* ^2^	*P*
Age (years old)	65.85 ± 15.01	68.70 ± 13.11	64.72 ± 11.54	1.650	0.195
Sex (male)	13 (39.40%)	28 (56.00%)	67 (67.00%)	8.077	0.018

Tha: thalassemia, IDA: iron-deficiency anemia, NA: non-anemia.

Platelet counts in the Tha, NA, and IDA groups were different and statistically significant (*P* < 0.05). Table 2 compares the platelet count among the three groups.

**Table 2:** Comparison of platelet count among the three groups

Variables	Tha (*n* = 33)	IDA (*n* = 50)	NA (*n* = 100)	*F*	*P*
PLT (×10^9^/L)	301.61 ± 29.22	261.4 ± 96.45#	241.46 ± 64.95#	9.060	<0.001

#: compared with the Tha group, *P* < 0.05.

The average Hb concentration in the Tha group was 83.73 ± 18.63 g/L. The average Hb concentration in the anemia group was 92.6 g ± 16.53 g/L. Compared with the Tha group, the IDA group had a lower proportion of patients with moderate anemia and a higher proportion of patients with mild anemia (*P* < 0.05). Table 3 compares the anemia indices between the Tha and IDA groups.

**Table 3:** Comparison of anemia indices between the Tha and IDA groups

Variables	Tha (*n* = 33)	IDA (*n* = 50)	*χ* ^2^/*t*	*P*
Sex(male/female)	13/20	28/22	2.193	0.139
Mild anemia	11 (33.3%)	34 (68.0%)	13.207	<0.001
Moderate anemia	18 (54.5%)	14 (28.0%)	5.913	0.015
Severe anemia	4 (12.1%)	2 (4.0%)	0.932	0.334
Average	83.73 ± 18.63	92.6 ± 16.53	2.274	0.027

### 3.2 Analysis of risk factors for cerebral infarction in the three groups

The prevalence of hypertension in the Tha group was lower than that in the other two groups, and the difference was statistically significant (*P* < 0.05). There were significantly fewer 0–2 risk factors of cerebral infarction in the Tha and IDA groups than those in the NA group (*P* < 0.05). The relationship between the risk factors of cerebral infarction and Tha and IDA in the three groups is shown in Table 4.

**Table 4:** Association between risk factors of cerebral infarction and thalassemia and IDA

Variables	Tha (*n* = 33)	IDA (*n* = 50)	NA (*n* = 100)	*χ* ^2^/*F*	*P*
High blood pressure	15 (45.5%)	33 (66.0%)	69 (69.0%)	6.093	0.048
Diabetes	11 (33.3%)	14 (28.0%)	25 (25.0%)	0.884	0.643
Hyperlipidemia	19 (57.6%)	23 (46.0%)	69 (69.0%)	5.832	0.501
Hcy	1 (3.0%)	7 (14.0%)	11 (11.0%)	2.614	0.273
Smoking history	7 (21.2%)	11 (22.0%)	36 (36.0%)	4.473	0.107
Drinking history	2 (6.0%)	6 (12.0%)	18 (18.0%)	2.930	0.235
Carotid arteriosclerosis/carotid stenosis	28 (84.8%)	46 (92.0%)	91 (91.0%)	1.391	0.527
Intracranial arteriosclerosis or stenosis	27 (81.9%)	45 (90.0%)	91 (91.0%)	2.217	0.352
0–2 risk factors	19 (57.6%)	36 (72.0%)	42 (42.0%)	12.381	0.002

### 3.3 Statistical analysis of clinical symptoms and signs in the three groups

There was no significant difference in clinical symptoms or signs among the three groups (*P* > 0.05). Table 5 compares the neurological symptoms or signs among the three groups.

**Table 5:** Comparison of the neurological symptoms or signs among the three groups

Variables	Tha (*n* = 33)	IDA (*n* = 50)	NA (*n* = 100)	*χ* ^2^/*F*	*P*
Disturbance of consciousness	3 (9.1%)	6 (12.0%)	3 (3.0%)	0.286	0.07
Abnormal feeling	8 (24.2%)	11 (22.0%)	20 (20.0%)	0.448	0.22
Movement disorder	30 (90.9%)	44 (88.0%)	86 (86.0%)	0.342	0.96
Speech disorder	11 (33.3%)	19 (38.0%)	39 (39.0%)	5.063	0.81
Ataxia	4 (12.1%)	8 (16.0%)	13 (13.0%)	0.384	0.89
Cognitive impairment	1 (3.0%)	4 (8.0%)	5 (5.0%)	0.966	0.66
Mental disorder	0 (0.0%)	2 (4.0%)	0 (0.0%)	3.794	0.10
Visual impairment	1 (3.0%)	1 (2.0%)	3 (3.0%)	0.381	0.92

## 4 Distribution and size of infarcts

All patients underwent brain MRI, and it was found that the cerebral infarction lesions in the Tha and IDA groups were mainly located in the corona radiata and centrum semiovale, and the cerebral infarction lesions in the NA group were more frequent in the basal ganglia area. There was a statistically significant difference in the lesion site between the Tha and NA groups (*P* < 0.05), while there was no significant difference between the Tha and IDA groups (*P* > 0.05). Statistical analysis of the infarct size in the three groups was performed, and no statistically significant difference was found in lesion size among the three groups (*P* > 0.05). In addition, it was attempted to indicate whether the three groups of patients had watershed cerebral infarctions, the vascular distribution of infarcts, and multiple lesions. It was revealed that the lesions were mainly involved in the anterior circulation in the three groups, and there was no significant difference among the three groups (*P* > 0.05). There was a high incidence of watershed infarction in the Tha group compared with that in the IDA group. The infarct lesions in the Tha and IDA groups were mainly multiple-type, and there was a significant difference in the prevalence of infarct lesions between the Tha and NA groups (*P* < 0.05). Compared with the NA group, the infarct lesions in the IDA and Tha groups were more frequently identified in corona radiata and centrum semiovale (*P* < 0.05). The imaging characteristics of infarct lesions in the three groups are presented in Table 6.

**Table 6:** Imaging characteristics of infarct lesions in the three groups

Variables	Tha (*n* = 33)	IDA (*n* = 50)	NA (*n* = 100)	*χ* ^2^/*F*	*p*
Anterior circulation	21 (63.6%)	31 (62.0%)	65 (65.0%)	0.132	0.936
Posterior circulation	9 (27.3%)	15 (30.0%)	25 (25.0%)	0.430	0.807
Anterior circulation and posterior circulation	3 (9.1%)	4 (8.0%)	10 (10.0%)	0.179	0.942
Solitary	13 (39.4%)	18 (36.0%)	65 (65.0%)	3.129	0.220
Multiple	20 (60.6)	28 (64.0%)	35 (35.0%)	—	—
Small infarction	17 (51.5%)	26 (52.0%)	42 (42.0%)	1.756	0.416
Middle infarction	14 (42.4%)	20 (40.0%)	52 (52.0%)	2.265	0.322
Large infarction	2 (6.1%)	4 (8.0%)	6 (6.0%)	0.391	0.916
Cerebral watershed infarction	5 (15.2%)	6 (12.0%)	3 (3.0%)	7.379	0.002
Lobar infarction	3 (9.1%)	15 (30.0%)	33 (33.0%)	7.859	0.023
Corpus callosum infarction	0 (0.0%)	10 (20.0%)	11 (11.0%)	8.472	0.011
Basal ganglia infarction	13 (39.4%)	27 (54.0%)	56 (56.0%)	2.809	0.245
Cerebellar infarction	4 (12.1%)	6 (12.0%)	5 (5.0%)	3.326	0.196
Brain stem infarction	6 (18.2%)	10 (20.0%)	23 (23.0%)	0.414	0.813
Corona radiata and centrum semiovale infarction	14 (42.4%)	29 (58.0%)	27 (27.0%)	13.859	0.001

**Figure 1:**
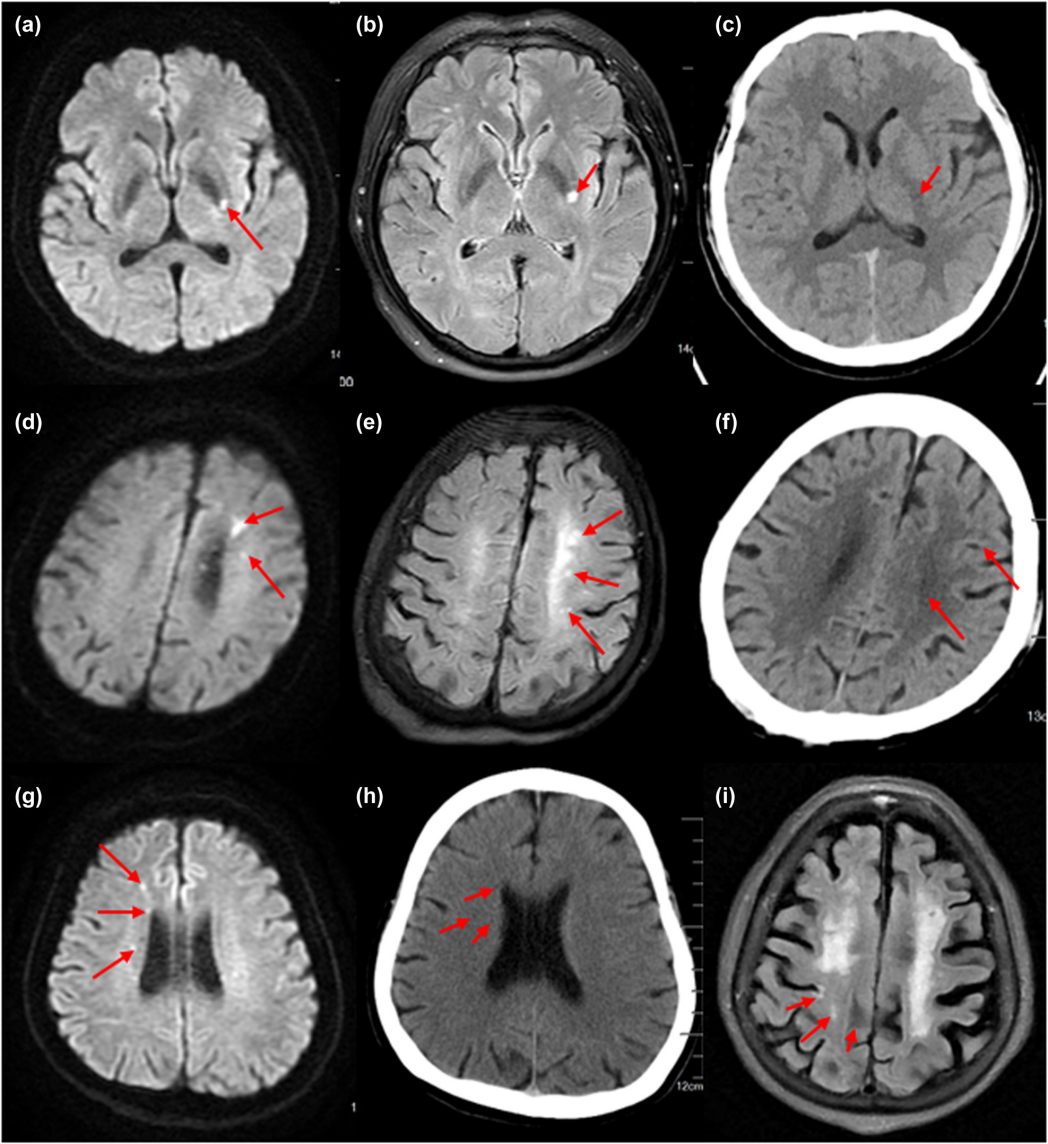
(a–c) In the NA group, single small infarcts in the basal ganglia were commonly identified. (d–f) In the Tha group, multiple small infarcts in the hemianopia and radial corona were commonly identified. (g–i) In patients with IDA combined with ACI, multiple small infarcts in the hemianopia and radial corona were also commonly identified. Patients with IDA combined with ACI also showed multiple small infarcts in the radial crown and semi-oval center.

In the NA group, single small infarcts in the basal ganglia were commonly identified (Figure 1a–c shows patient No. 25 on MRI-DWI, T2FLAIR, and CT axial images); in the Tha group, multiple small infarcts in the hemianopia and radial corona were commonly identified (Figure 1d–f shows patient No. 53 on MRI-DWI, T2FLAIR, and CT cranial images); in patients with IDA combined with ACI, multiple small infarcts in the hemianopia and radial corona were also commonly identified. Patients with IDA combined with ACI also showed multiple small infarcts in the radial crown and semi-oval center (Figure 1g and h shows patient No. 36 on MRI-DWI and cranial CT axial images, Figure 1i shows patient No. 37 on MRI-T2 axial images).

## 5 Discussion

In recent years, the aging of China’s population has been intensified, and the incidence of anemia is also increasing year by year. Anemia is closely associated with human health. Therefore, the relationship between anemia and ACI has gradually attracted scholars’ attention. Studies have confirmed that some types of anemia are closely correlated with ACI, such as sickle cell anemia (SCA), blood loss anemia, and IDA, of which SCA and IDA are worthy of further assessment. Chronic anemia is closely related to childhood cerebral infarction, mostly asymptomatic watershed cerebral infarction, and it is mainly associated with large artery stenosis or occlusion [8]. In recent years, some scholars have found that patients with anemia ACI often lack traditional common risk factors and are at a greater risk of experiencing clinical disturbance of consciousness. MRI is the most appropriate method to detect early signs of cerebral ischemia and intracranial hemorrhage. Besides, watershed infarctions are more prone than other cortical infarcts to cause early-onset seizures [7,9]. These studies confirmed that ACI patients with anemia have unique clinical characteristics, and anemia is closely associated with ACI.

### 5.1 Current status of research on ACI patients with thalassemia

With the improvement of medical services, the average life expectancy of thalassemia patients has been prolonged, while relevant complications have also emerged. The incidence of asymptomatic cerebral infarction has been reported to be higher in children with thalassemia [10] compared with that in adults [11], especially in patients with splenectomy; thalassemia patients with splenectomy are prone to stenosis or occlusion of large intracranial arteries [12]. ACI occurred in 0.25% of patients with β-thalassemia [13], and some scholars demonstrated that ACI occurred in two patients with TI and five patients with TM through case reports. All patients had splenectomy, and the average Hb level was 7.5 g/L. Among them, there were five cases of regular blood transfusion and one case of left internal carotid artery stenosis [14]. Another study reported a 25-year-old female case of TM with infarction in the left corona radiata and centrum semiovale, in which the average Hb level was 7 g/L, and the left internal carotid artery was occluded [15].

In the present study, the clinical characteristics of ACI patients with thalassemia were analyzed. As the most common type of cerebrovascular disease, the number of patients with ACI continues to increase every year, with an average rate of 8.7% per year [6]. A previous epidemiological survey showed that the incidence rates of α-thalassemia, β-thalassemia, and α + β-thalassemia in the reproductive age population in Hainan province were 10.39, 1.38, and 1.18%, respectively [16]. Another study demonstrated that the hypercoagulable state of patients with β-thalassemia might lead to thromboembolic events in multiple organs, including brain involvement [17], while the relevant clinical characteristics of ACI patients with thalassemia have been scarcely reported.

Thalassemia, as an autosomal single-gene hereditary disease, has no significant difference in the incidence rate between men and women, while there are more female patients than male patients in some epidemiological surveys [4]. The present study also reached the same conclusion, which may be related to the promotion of prenatal diagnosis of thalassemia, as well as the significant regional differences in thalassemia and other factors. It is noteworthy that Wang et al.’s results showed that men who aged >40 years old had a higher incidence of ACI than women [18], which was different from other findings reported previously. Therefore, whether the occurrence of ACI with thalassemia is correlated with gender needs to be further studied by expanding the sample size.

Thalassemia results from inherited abnormalities of the synthesis of the globin chains of Hb. Eldor et al. [19] studied thalassemia patients and found that platelet aggregation in peripheral blood of thalassemia patients significantly increased, platelet destruction was elevated, and average lifespan was shortened. Some studies have shown that there are higher concentrations of thromboxane A2 metabolites, thromboxane B26, and 6‐keto‐prostaglandin Flα in the urinary samples of patients with thalassemia, and they are mainly the strongest vasoconstrictors and platelet aggregation agents. It was found that the expression levels of platelet activation markers (CD62P and CD63) were significantly upregulated in patients with thalassemia [20,21]. The results of the present study suggested that both the Tha group and the IDA group were in a hypercoagulable state with secondary thrombocytosis compared with the NA group during the same period, which was consistent with Karimi et al.’s results [14].

A prospective survey showed that 90% of 478 ACI patients had more than 2 risk factors for cerebrovascular disease [22], while in the present study, 19 (57.6%) patients in the Tha group had 0–2 risk factors for cerebral infarction, and there were 14 (42.4%) cases with more than 2 risk factors; in the NA group, 42 (42.0%) cases had 0–2 risk factors for cerebral infarction and 58 (58.0%) cases had more than 2 risk factors; in the IDA group, 36 (72.0%) cases had 0–2 risk factors for cerebral infarction and 14 (28.0%) cases had more than 2 risk factors. This study also indicated that there was no significant difference in clinical neurological symptoms or signs between the Tha group and the other two groups (*P* > 0.05). There were 3 (9.1%) patients with disturbance of consciousness in the Tha group.

### 5.2 Shortcomings

When anemia occurs in ACI patients, the demand for oxygen in the brain increases, and at this time, the oxygen-carrying capacity of the blood decreases, resulting in insufficient energy of neurons, causing local or comprehensive neurological dysfunction. The blood vessels in the anterior circulation occupy two-thirds of the blood supply of the brain and naturally require more oxygen; the anterior circulation is at a higher position in the brain, and the areas supplied by the anterior circulation are relatively tolerant to ischemia and hypoxia. Therefore, it is more susceptible to anemia, and the blood supply and oxygen supply are insufficient, resulting in the occurrence of infarct lesions; the watershed area, corona radiata, and centrum semiovale are generally dominated by terminal cerebral vessels, and collateral vessels are not abundant. Therefore, when anemia occurs, these areas have a high probability of hypoperfusion. If anemia is not timely treated, it may eventually develop into an infarction [23,24]. The results of the present study indicated that lesions in the Tha and IDA groups were mainly located in the region from the corona radiata and the centrum semiovale, in which multiple small infarcts were dominant, and there was no significant difference between the two groups (*P* > 0.05). In the NA group, patients’ lesions were mainly found in the basal ganglia area, and single small infarcts were dominant, which were significantly different from those observed in the Tha and IDA groups (*P* < 0.05). However, there are some differences between our findings and previously reported results. Comparing the brain MRI examination of thalassemia patients with early clinical manifestations of a series of neurological complications, it was found that thalassemia patients had infarction in the putamen, caudate nucleus, and cortex, and an abnormal iron deposition was identified in these areas. Thus, it can be speculated that infarction in patients with thalassemia is more likely to occur in the putamen, caudate nucleus, and cortex [25]. Therefore, the intracranial infarct lesions in patients with thalassemia are worthy of further investigation, and additional large-scale studies should be conducted to confirm the findings of the present study.
